# Surgical site infections and the discharge care of surgical drains following spinal fusions: a qualitative inquiry

**DOI:** 10.1017/ash.2025.10152

**Published:** 2025-10-01

**Authors:** Sydney LeSon, Jean Horoho, Leonard Mermel

**Affiliations:** 1 Department of Epidemiology and Infection Prevention, Brown University Health, Providence, RI, USA; 2 Division of Infectious Diseases, Department of Medicine, Warren Alpert Medical School of Brown University Providence, Providence, RI, USA

## Abstract

Patient interviews suggest there is a difference in surgical drain management after hospital discharge among patients who developed surgical site infection (SSI) compared to those without an SSI. Based on our findings, postoperative drain care instruction was revised with the goal of improving compliance with management of this device.

## Introduction

Infection prevention surveillance at Rhode Island Hospital revealed many patients with surgical site infections (SSIs) after neurosurgical spinal fusion procedures were discharged with surgical drains. Prolonged surgical drain retention is associated with risk of SSIs.^1^ Little is known about drain management among patients after hospital discharge.

Deep surgical drains are placed during spine surgery to control postsurgical wound drainage and reduce the risk of seroma and hematoma developing in the surgical bed. A literature review determined that the use of spinal drains following spinal surgery showed no consistent evidence supporting a significant reduction in infection or hematoma rates.^2^ Moreover, a retrospective study found surgical drain duration was independently associated with risk of SSI (OR 1.36 per day of drain placement; *P* = 0.02).^1^

A systematic review concluded “Insufficient evidence has been published thus far to conclude whether the placement of supra- or sub-fascial drains is beneficial following spinal surgery.”^3^ and authors of a retrospective study of patients who underwent spinal fusion concluded “No significant differences in wound infection rates were noted between patients with and without drains (3.5% vs 2.6%, *P* = 0.6).”^4^ Regarding patients discharged after spine surgery, another study concluded that “Patients may be safely discharged from the hospital with the surgical drain in place.”^5^

## Objective

We set out to better understand current care practices for post spinal fusion drains after hospital discharge to determine if there is a difference in drain management in patients who developed SSIs compared to those without such infections.

## Methods

Patients who had spinal fusion by any neurosurgeon April 1, 2022—April 30, 2023 at our tertiary care hospital and who were discharged with a surgical drain were eligible for inclusion in this IRB-approved study. The surveillance period for an SSI was 90 days and included deep and organ space SSIs. Drains were closed self-suction. Twenty-four of 28 neurosurgery patients who met the eligibility criteria and who developed an SSI were discharged with a surgical drain. We randomly selected 24 patients who had spinal fusion by a neurosurgeon during the same time frame and who did not develop an SSI to serve as controls.

We attempted to contact these 48 patients January 2024—March 2024 regarding their experience and knowledge when caring for their surgical drain after hospital discharge. No patient included in this study was discharged with a known SSI. Patients were contacted and interviewed by phone without compensation (Table 1).

**Table 1.** Telephone interview questions

Interview Questions
1. Did you go home or to a skilled nursing facility (SNF) after leaving the hospital with a drain? 2. Who took care of the drain? (Patient, family or friend, healthcare worker)* 3. What did you do to care for the drain? What steps did you take? 4. While you had the drain, did you take a shower, tub bathe, sponge bathe, or did not do either? If yes, how many times? 5. Did you do anything to the insertion site before showering or bathing? 6. Did you clean the drain insertion site besides showering/bathing? If so, with what? And how often? 7. Did you apply any dressings or ointment around the drain site? 8. Did your drain fall out at all, and how often? 9. How were you instructed to care for the drain before leaving the hospital? Did they provide any instruction regarding washing your hands or wearing gloves? 10. Overall, did you have any difficulty managing the drain?

*If patient stated another caregiver took care of the drain, the following questions were altered to “you or your caregiver.”

## Results

Eleven of 24 cases and 11 of 24 controls were successfully contacted and interviewed. Patients who had an SSI stated they experienced more difficulties managing their surgical drain after discharge compared to controls. For example, patients cited that because the surgical site was on their back it was difficult to reach behind them. If they did not have a caregiver or nurse helping them take care of the drain, they simply neglected care. Furthermore, these infected patients stated they received minimal discharge care instructions for their surgical drain or did not remember receiving any instructions, unlike the responses from controls. Several patients who were discharged to skilled nursing facilities did not actively care for their drain. Nurses took care of the drain but may not have had the discharge instructions to properly care for the drain.

8 of 11 patients without an SSI went home and the rest went to a skilled nursing facility (SNF). Of the patients who went home, 4 took care of the drain themselves. Of patients with an SSI, 5 of 11 went home. All 11 patients with an SSI, no matter where they were discharged, had another individual (family or nurse) manage their drain care. The mean drain duration for cases and controls was 17 days and 15 days, respectively (Table 2).

**Table 2.** Post-operative surgical drain duration

Drain Duration	Mean (Days)	St Dev. (Days)	Range (Days)	*P*-value
**Cases**	17	7.6	3–33	0.3
**Controls**	15	8.1	3–30	

Fewer patients with an SSI sponge bathed or showered compared to patients without an SSI. No patients from either group applied dressings or ointments to the drain site. Patients from both groups recalled emptying the drain reservoir when full; however, none of the infected patients remembered how frequently it was emptied or whether they washed their hands prior to emptying the drain or had worn gloves.

7 of the 11 patients without an SSI took care of their drain entirely by themselves or with the help of a partner or family member. All 7 patients remembered how often they emptied their drain reservoir or stated it was not emptied because the drain never became full. Additionally, these 7 patients recalled washing their hands prior to emptying the drain reservoir, with one citing they wore gloves.

Three patients who experienced an SSI noted that the drain tubing disconnected at least once, whereas only one patient without an SSI noted that their drain tubing disconnected once.

## Discussion

Patients who had an SSI experienced more difficulties managing surgical drains after hospital discharge compared to patients without an SSI. Since a greater proportion of patients with an SSI went to a SNF and none managed their own drains, these patients may have had more co-morbidities or underlying mental or physical limitations. Our interviews illustrated the significance in investigating patient experiences postdischarge to identify potential risk factors developing an SSI. Interviews with patients who experienced SSIs revealed more inconsistencies regarding their experiences, whereas patients who did not have SSIs recalled more straightforward experiences. Patients with an SSI stated they received minimal to no instruction regarding how to care for their drain. Based on our findings, we changed patient and caregiver education regarding drain care and ensured that instructions are provided in a consistent, easy to understand fashion.

Changes to drain care included the prioritization of hand hygiene when handling the drain, revised instructions for discharged patients, development of a supply kit of necessary items for emptying drains, and re-education of staff who provide discharge instructions to patients. Written instructions were reviewed by our neurosurgeons and plastic surgeons confirming consensus of proper bathing instructions and use of a chlorhexidine sponge dressing (Biopatch^TM^, Ethicon, Raritan, NJ) around the drain under the wound dressing to confirm consistent care after discharge.

If a patient is to be discharged to a SNF, nurses at the SNF are informed of updated instructions for proper drain management practices. Additionally, our infection control leadership has met with the neurosurgery staff to discuss limiting duration of drain use after hospital discharge.

This study took a novel approach to directly interview patients discharged with surgical drains to better understand how drains were managed; however, our study is not without limitations such as patients having recall bias. Lastly, the intent of the study was to hear in the patient’s own words how they managed their surgical drains so we set out to assess qualitative differences.

Patient interviews suggest there is a difference in surgical drain management after hospital discharge among patients with an SSI compared to patients without an SSI. Based on our findings, postoperative instructions were revised with the goal of improving compliance with drain management.

## Supporting information

10.1017/ash.2025.10152.sm001 Leson et al. supplementary material 1 Leson et al. supplementary material

10.1017/ash.2025.10152.sm002 Leson et al. supplementary material 2 Leson et al. supplementary material

## Supplementary material

The supplementary material for this article can be found at https://doi.org/10.1017/ash.2025.10152.
