# Supplementary material for: Surgical site infections and the discharge care of surgical drains following spinal fusions: a qualitative inquiry
Source: Antimicrob Steward Healthc Epidemiol. 2025 Oct 1;5(1):e241. doi: 10.1017/ash.2025.10152 (PMC12509140; doi:10.1017/ash.2025.10152)
Supplement: Leson et al. supplementary material 1 — Leson et al. supplementary material [file S2732494X25101526sup001.pptx]

## Slide 1
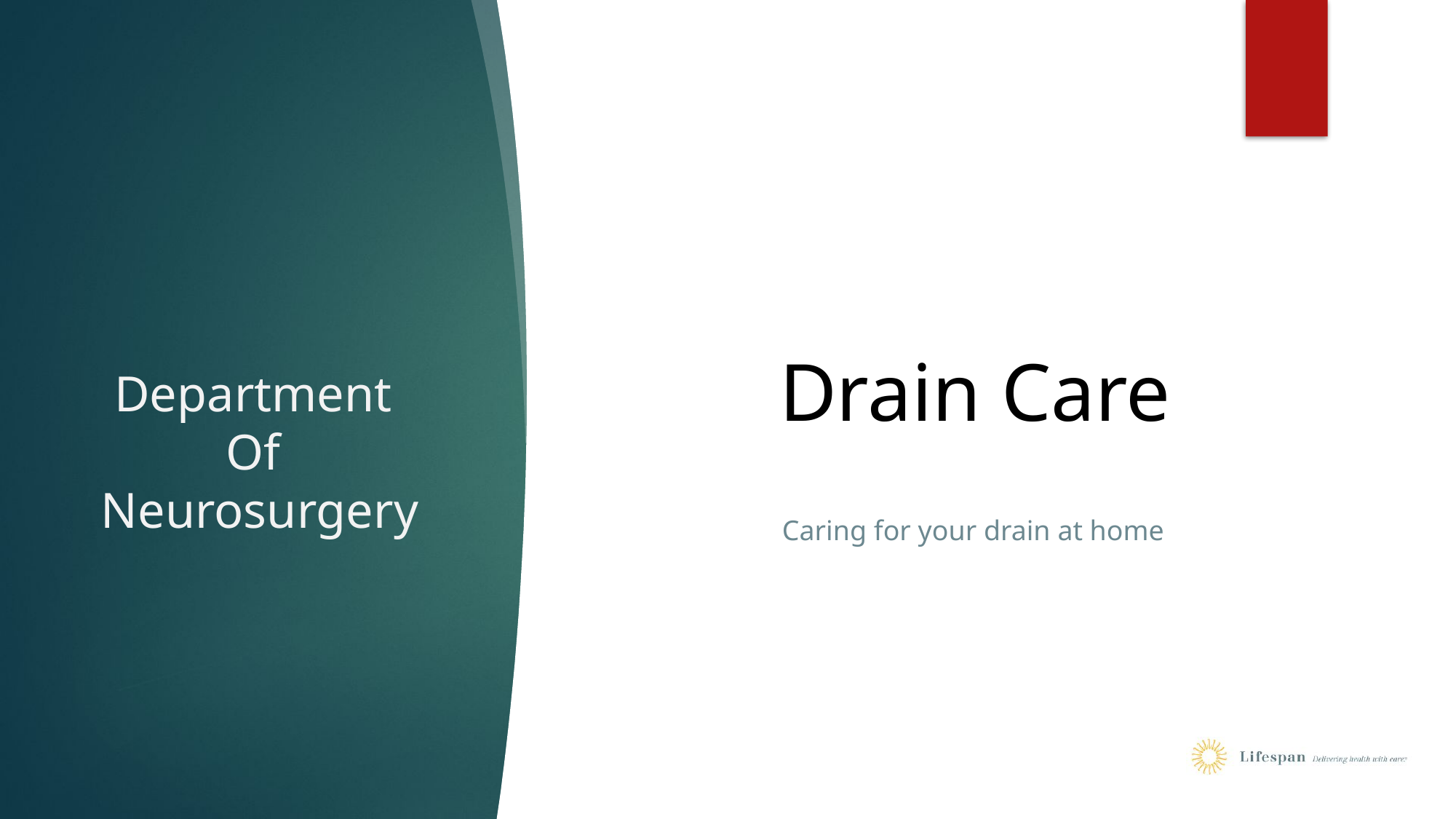

# Department Of Neurosurgery
Drain Care
Caring for your drain at home

## Slide 2
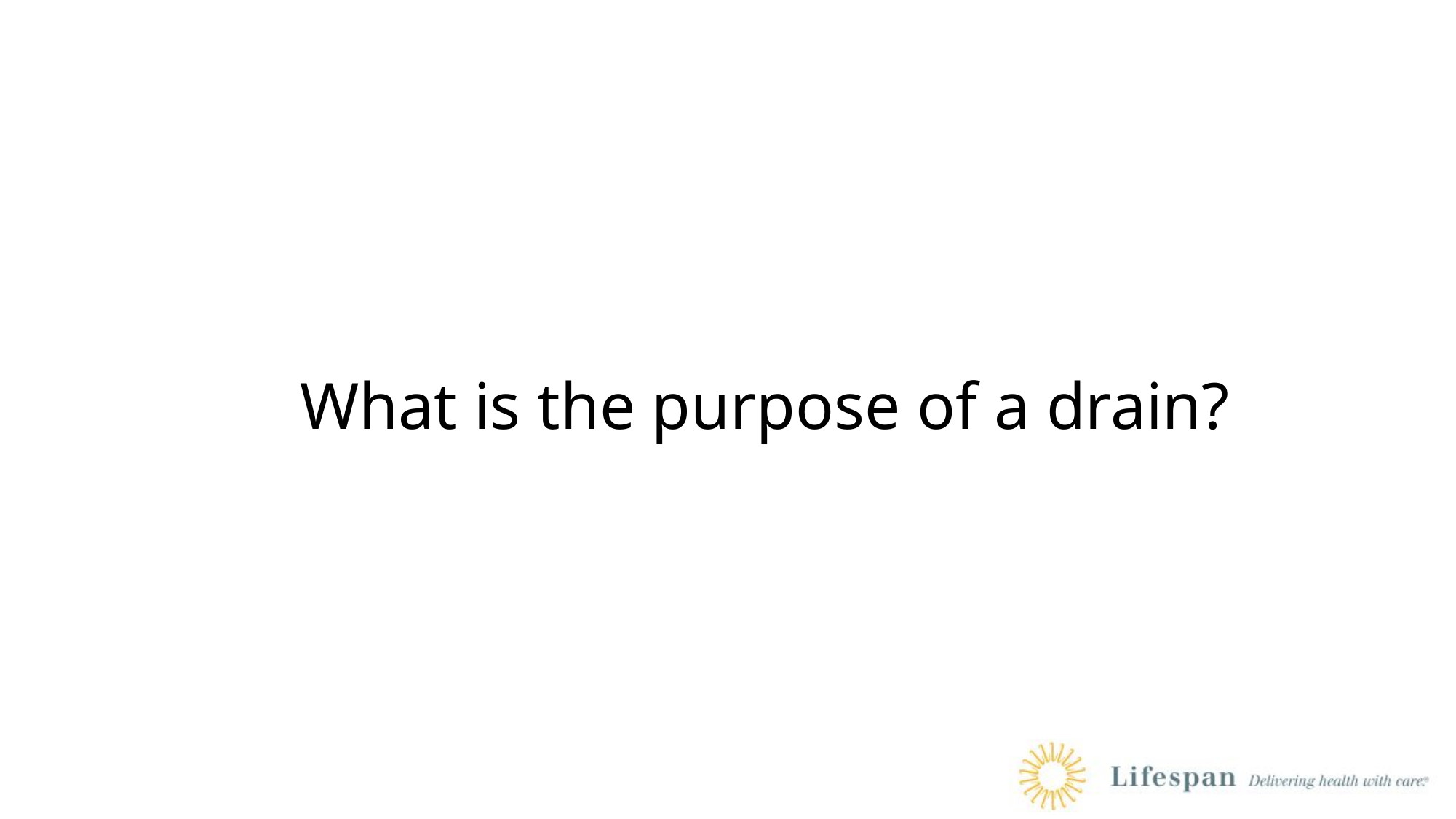

# What is the purpose of a drain?

## Slide 3
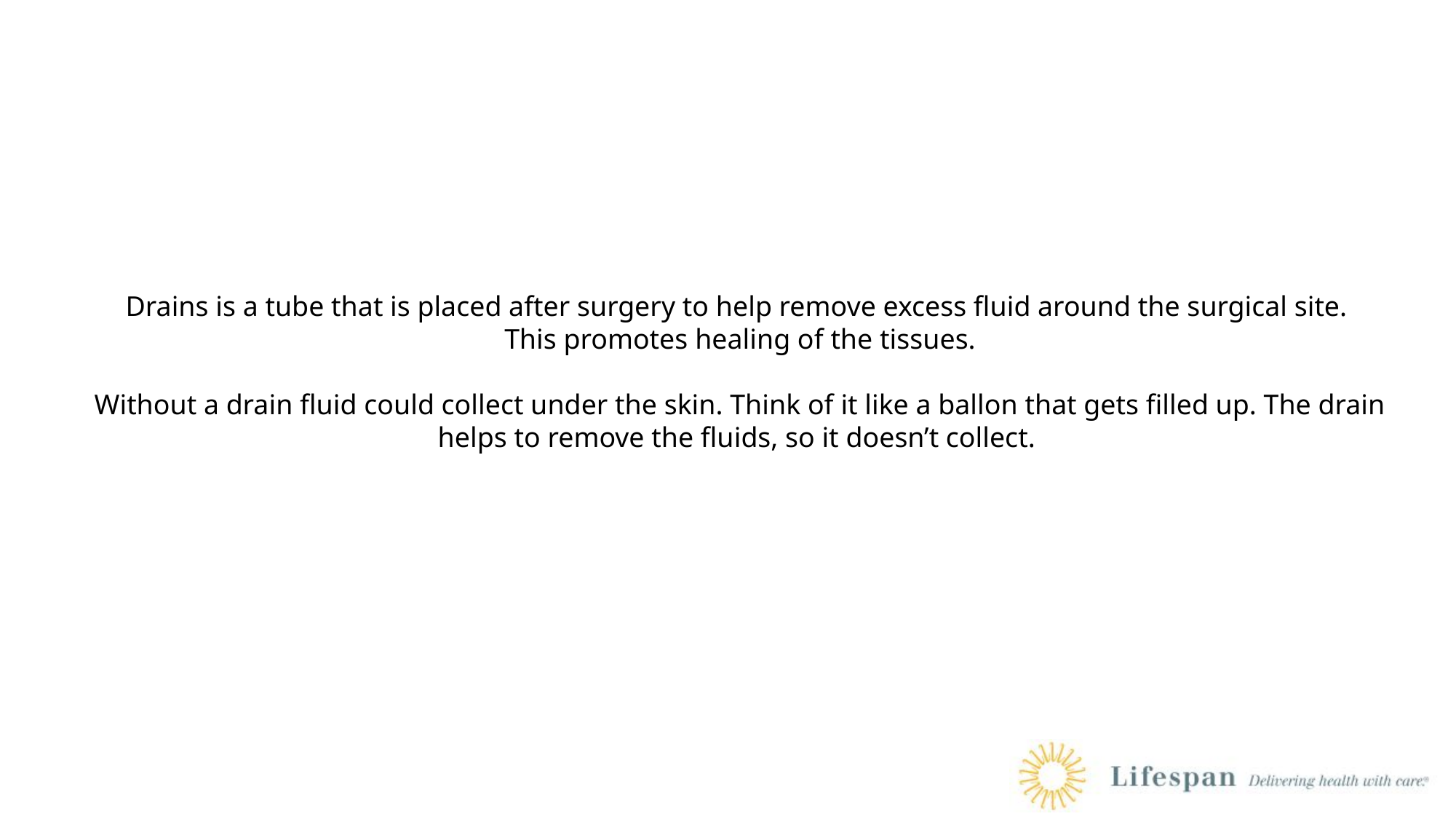

# Drains is a tube that is placed after surgery to help remove excess fluid around the surgical site. This promotes healing of the tissues. Without a drain fluid could collect under the skin. Think of it like a ballon that gets filled up. The drain helps to remove the fluids, so it doesn’t collect.

## Slide 4
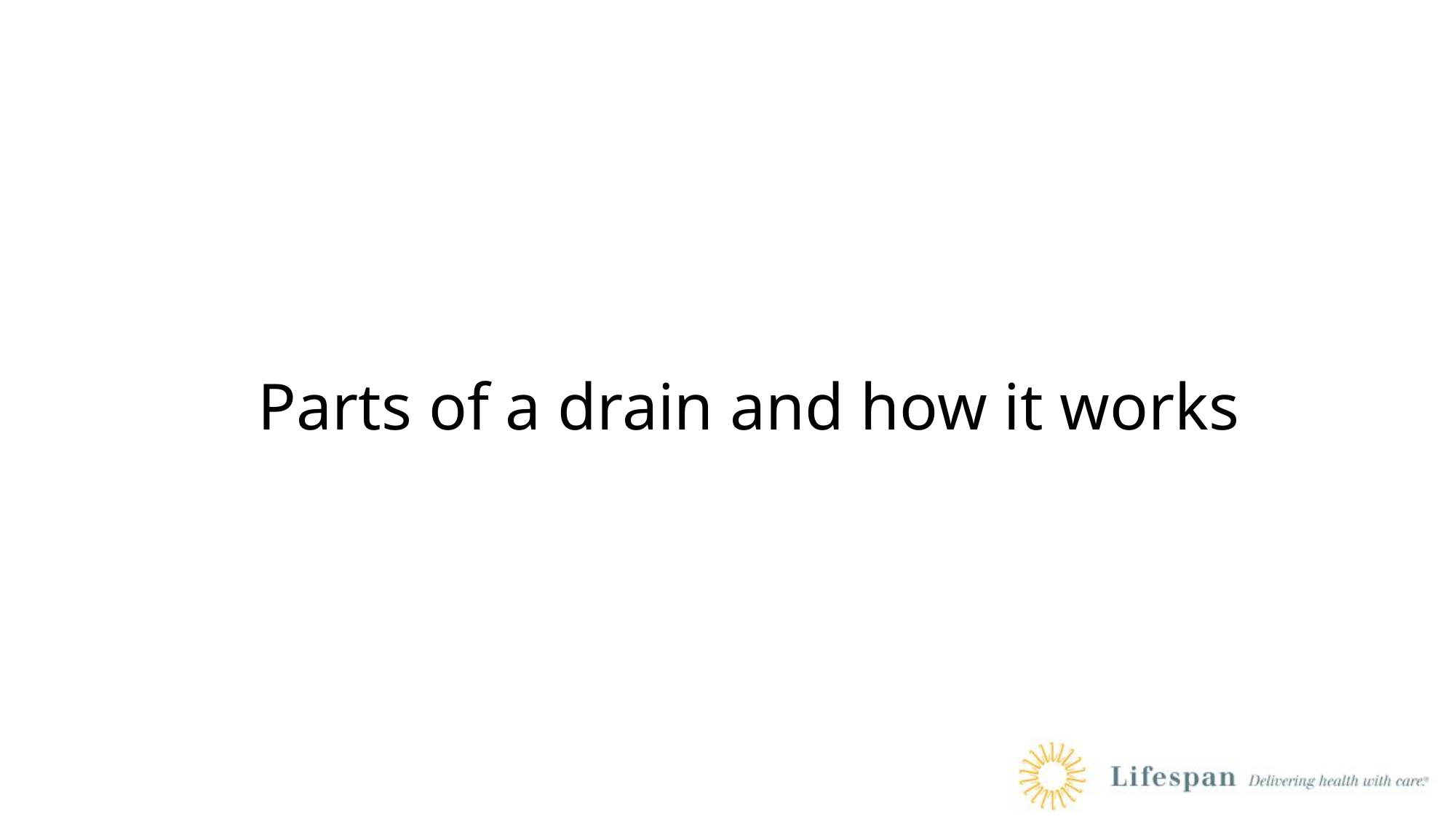

# Parts of a drain and how it works

## Slide 5
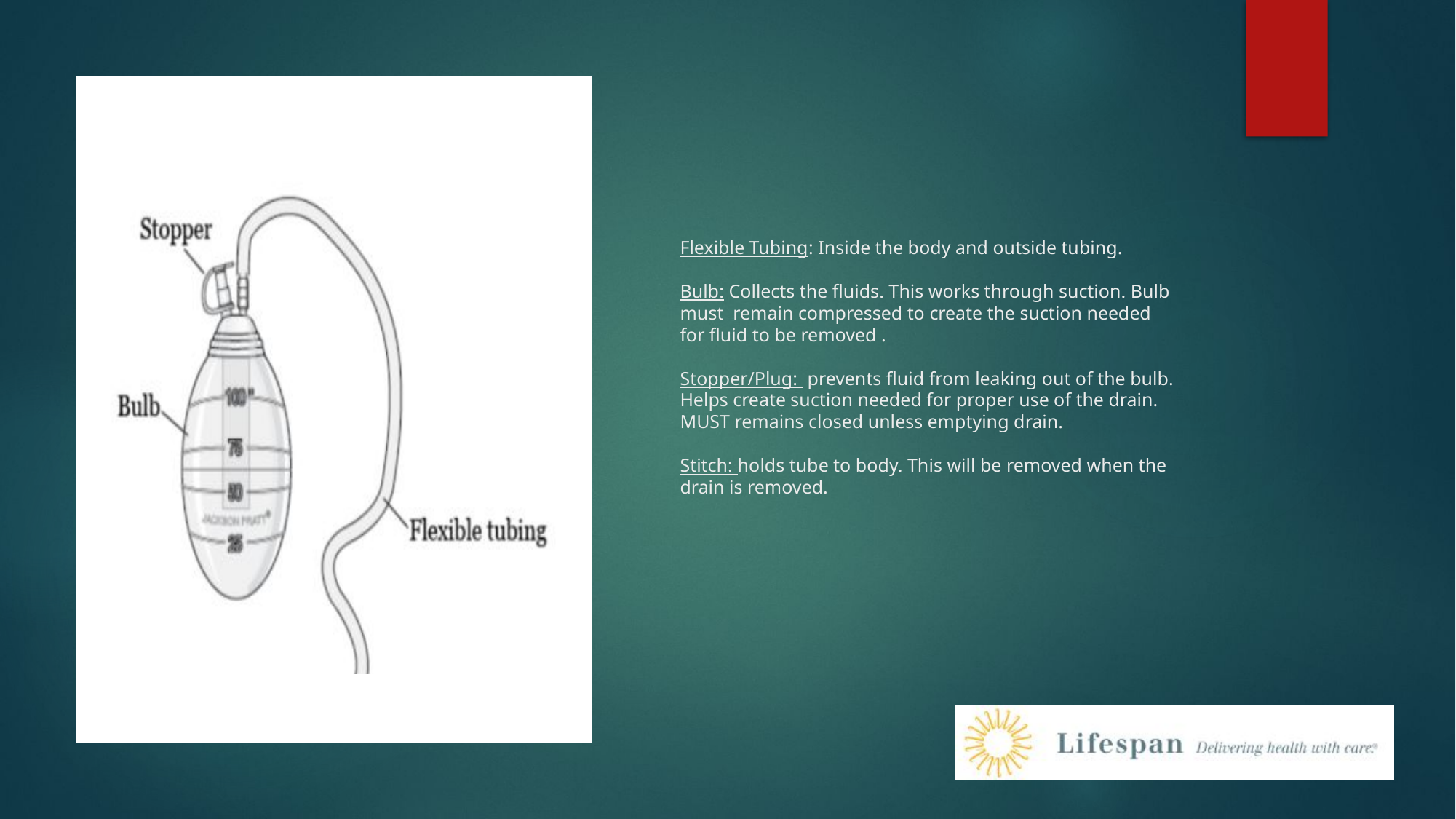

# Flexible Tubing: Inside the body and outside tubing. Bulb: Collects the fluids. This works through suction. Bulb must remain compressed to create the suction needed for fluid to be removed . Stopper/Plug: prevents fluid from leaking out of the bulb. Helps create suction needed for proper use of the drain. MUST remains closed unless emptying drain. Stitch: holds tube to body. This will be removed when the drain is removed.

## Slide 6
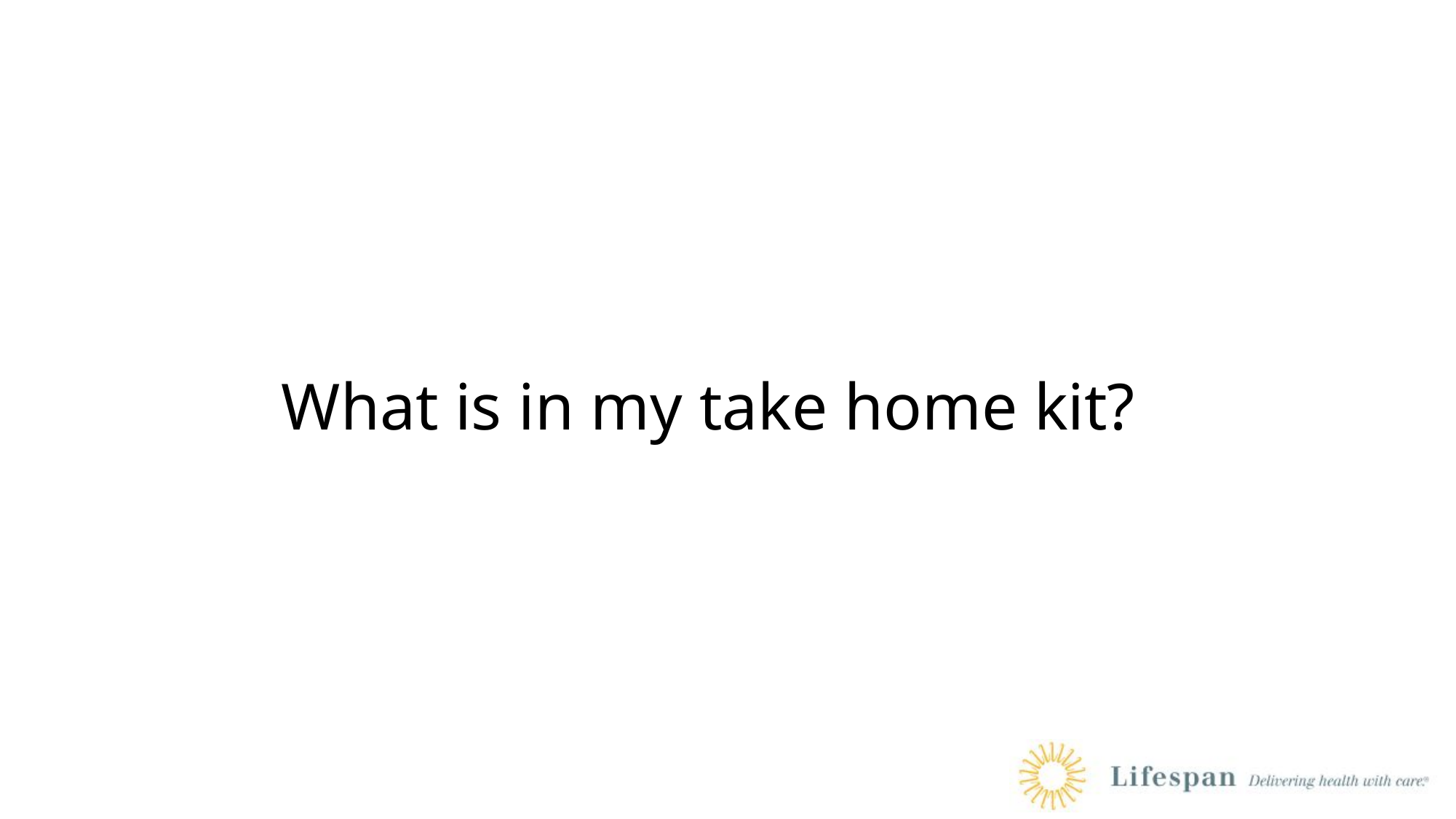

# What is in my take home kit?

## Slide 7
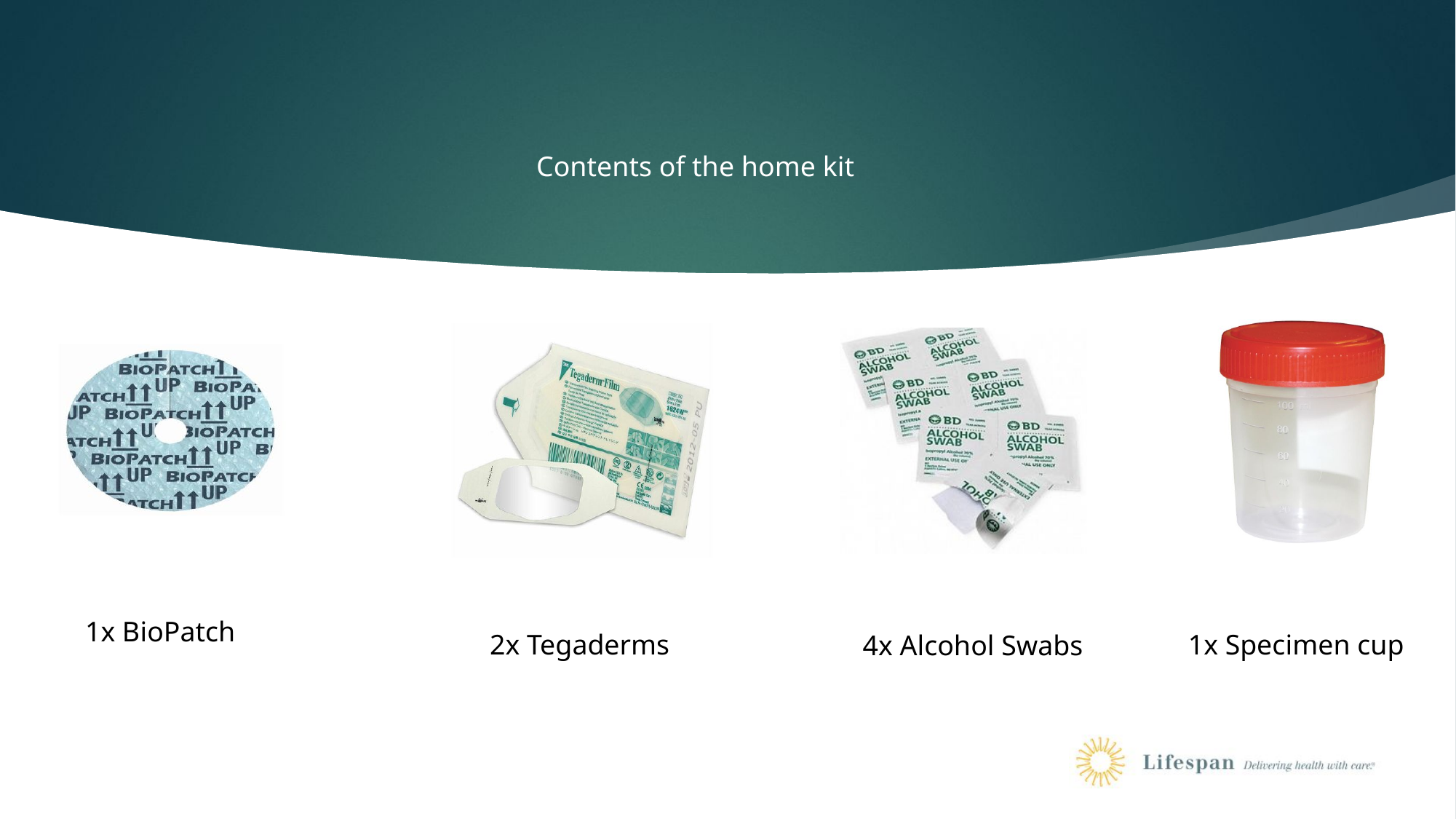

Contents of the home kit
11x BioPatch
2x Tegaderms
1x Specimen cup
4x Alcohol Swabs

## Slide 8
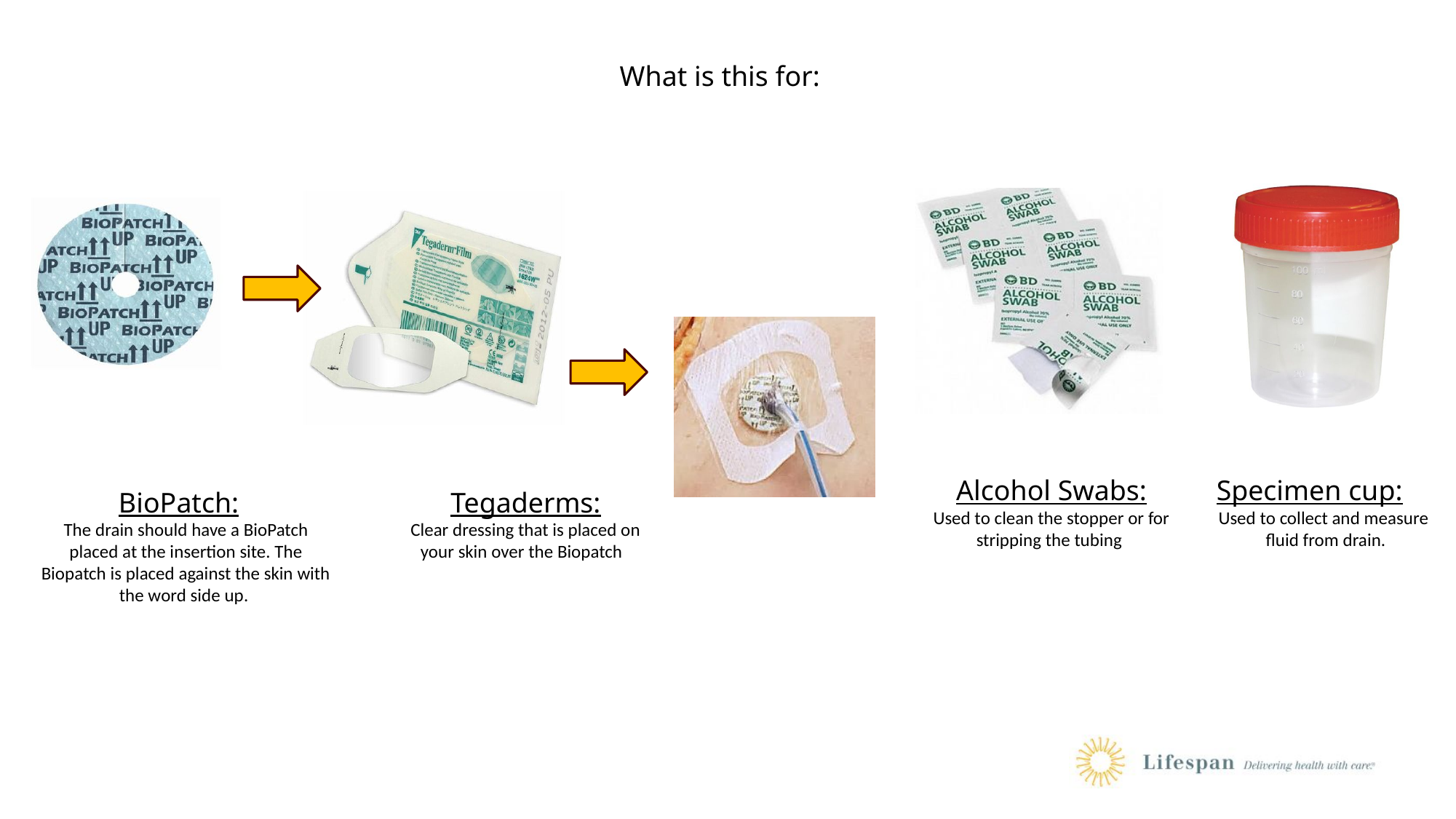

What is this for:
Contents of the home kit
Alcohol Swabs:
Used to clean the stopper or for stripping the tubing
Specimen cup:
Used to collect and measure fluid from drain.
1 BioPatch:
The drain should have a BioPatch placed at the insertion site. The Biopatch is placed against the skin with the word side up.
Tegaderms:
Clear dressing that is placed on your skin over the Biopatch

## Slide 9
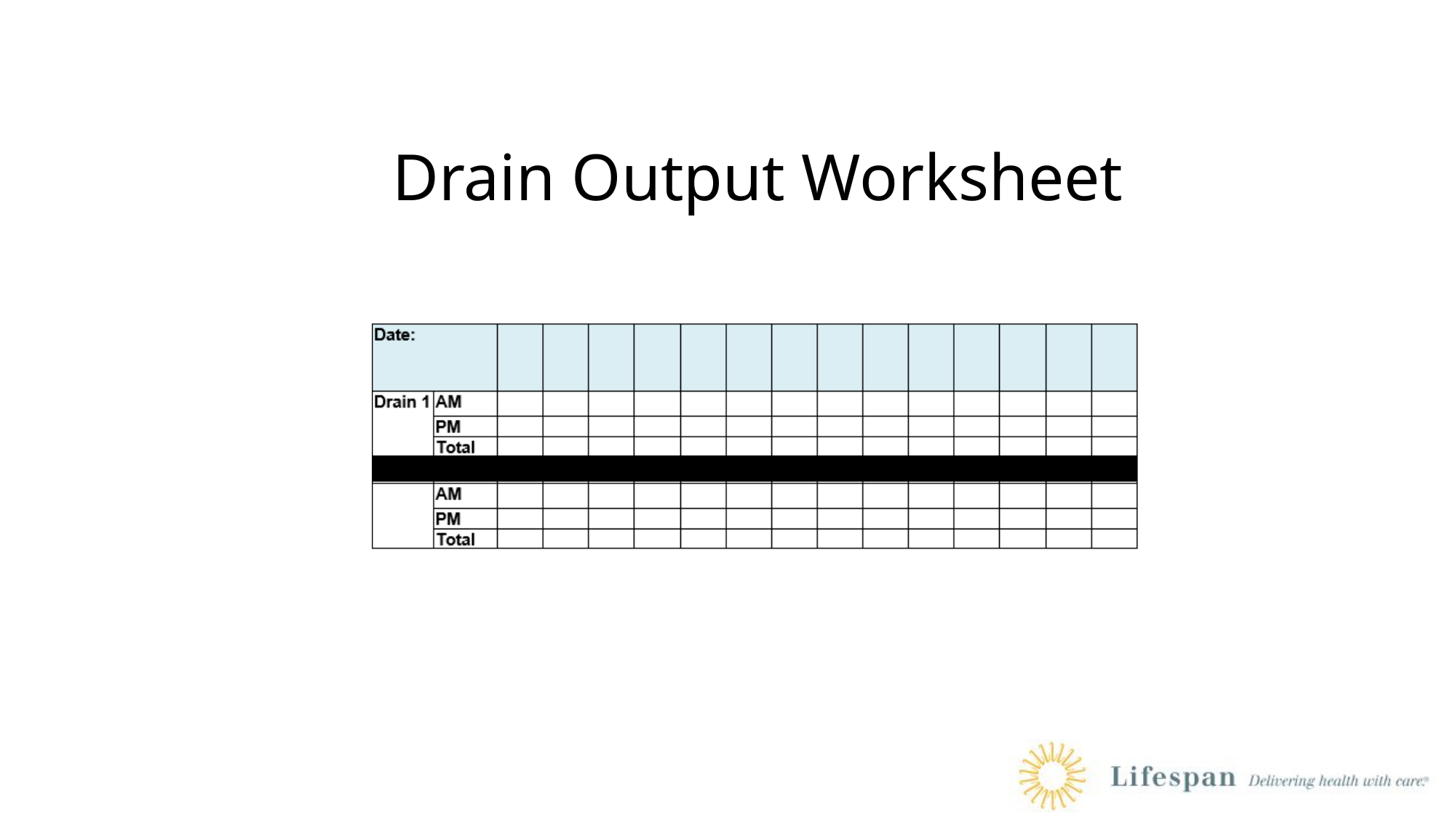

# Drain Output Worksheet

## Slide 10
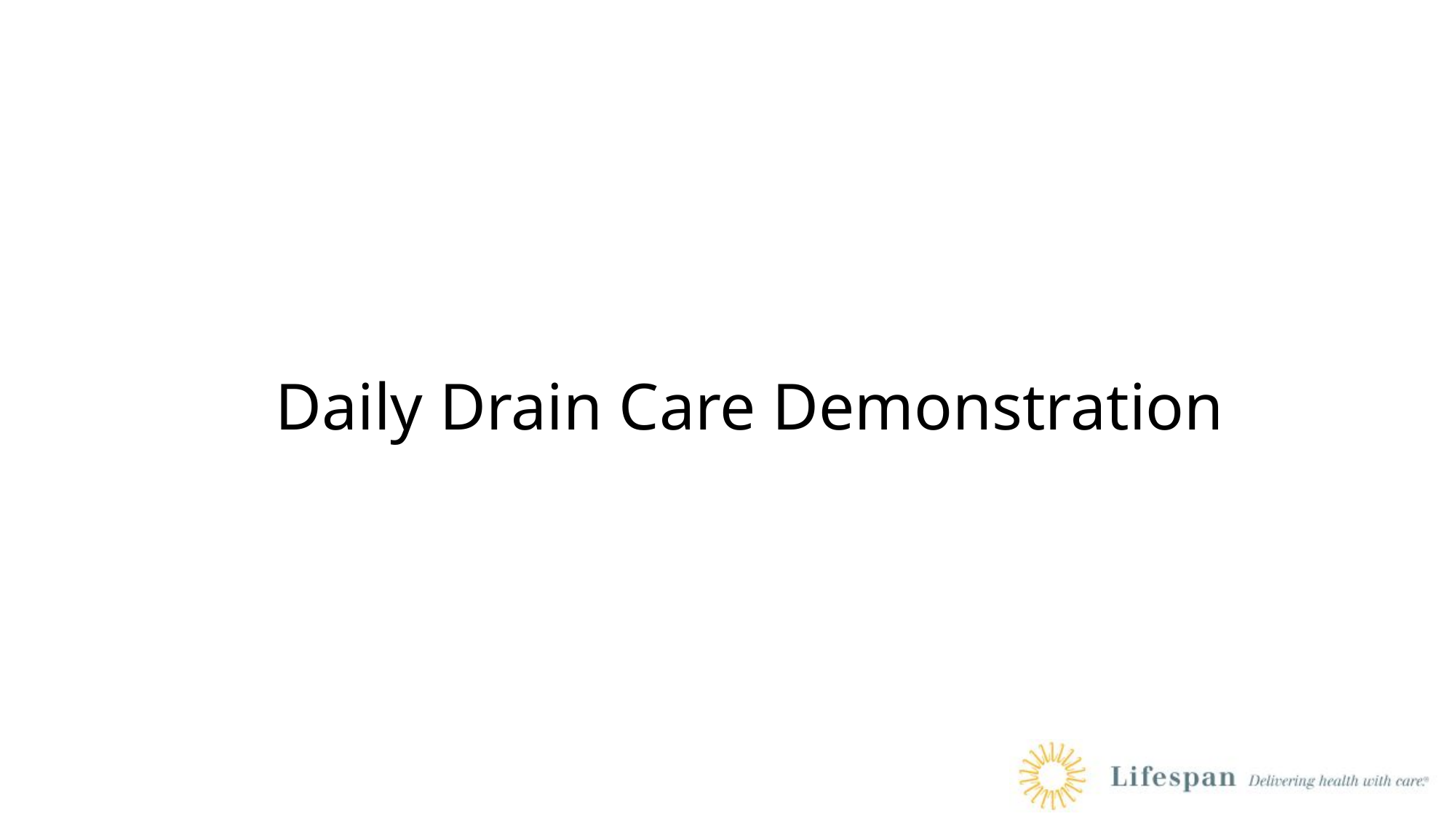

# Daily Drain Care Demonstration

## Slide 11
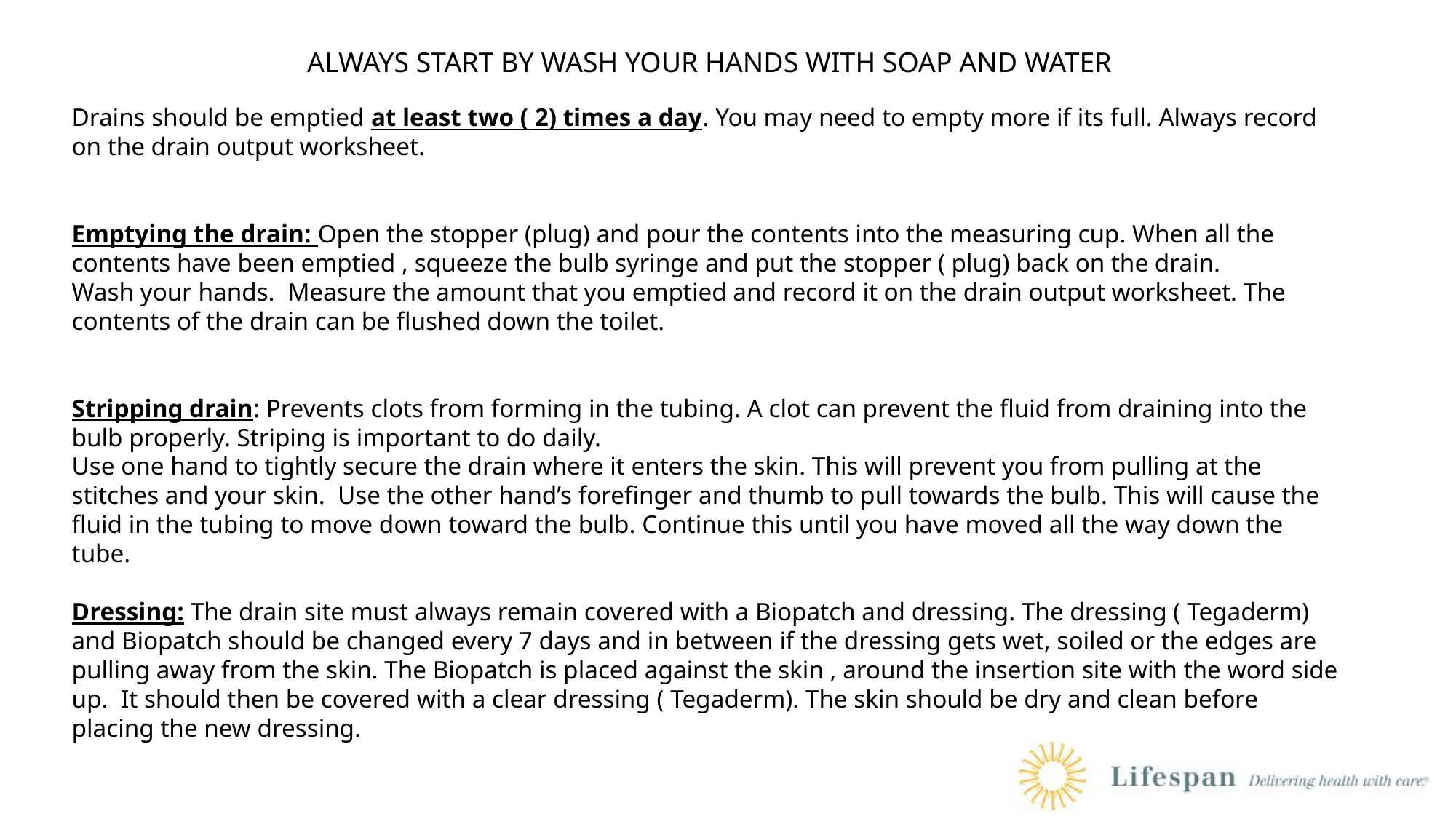

ALWAYS START BY WASH YOUR HANDS WITH SOAP AND WATER
# Drains should be emptied at least two ( 2) times a day. You may need to empty more if its full. Always record on the drain output worksheet. Emptying the drain: Open the stopper (plug) and pour the contents into the measuring cup. When all the contents have been emptied , squeeze the bulb syringe and put the stopper ( plug) back on the drain. Wash your hands. Measure the amount that you emptied and record it on the drain output worksheet. The contents of the drain can be flushed down the toilet. Stripping drain: Prevents clots from forming in the tubing. A clot can prevent the fluid from draining into the bulb properly. Striping is important to do daily. Use one hand to tightly secure the drain where it enters the skin. This will prevent you from pulling at the stitches and your skin. Use the other hand’s forefinger and thumb to pull towards the bulb. This will cause the fluid in the tubing to move down toward the bulb. Continue this until you have moved all the way down the tube. Dressing: The drain site must always remain covered with a Biopatch and dressing. The dressing ( Tegaderm) and Biopatch should be changed every 7 days and in between if the dressing gets wet, soiled or the edges are pulling away from the skin. The Biopatch is placed against the skin , around the insertion site with the word side up. It should then be covered with a clear dressing ( Tegaderm). The skin should be dry and clean before placing the new dressing.

## Slide 12
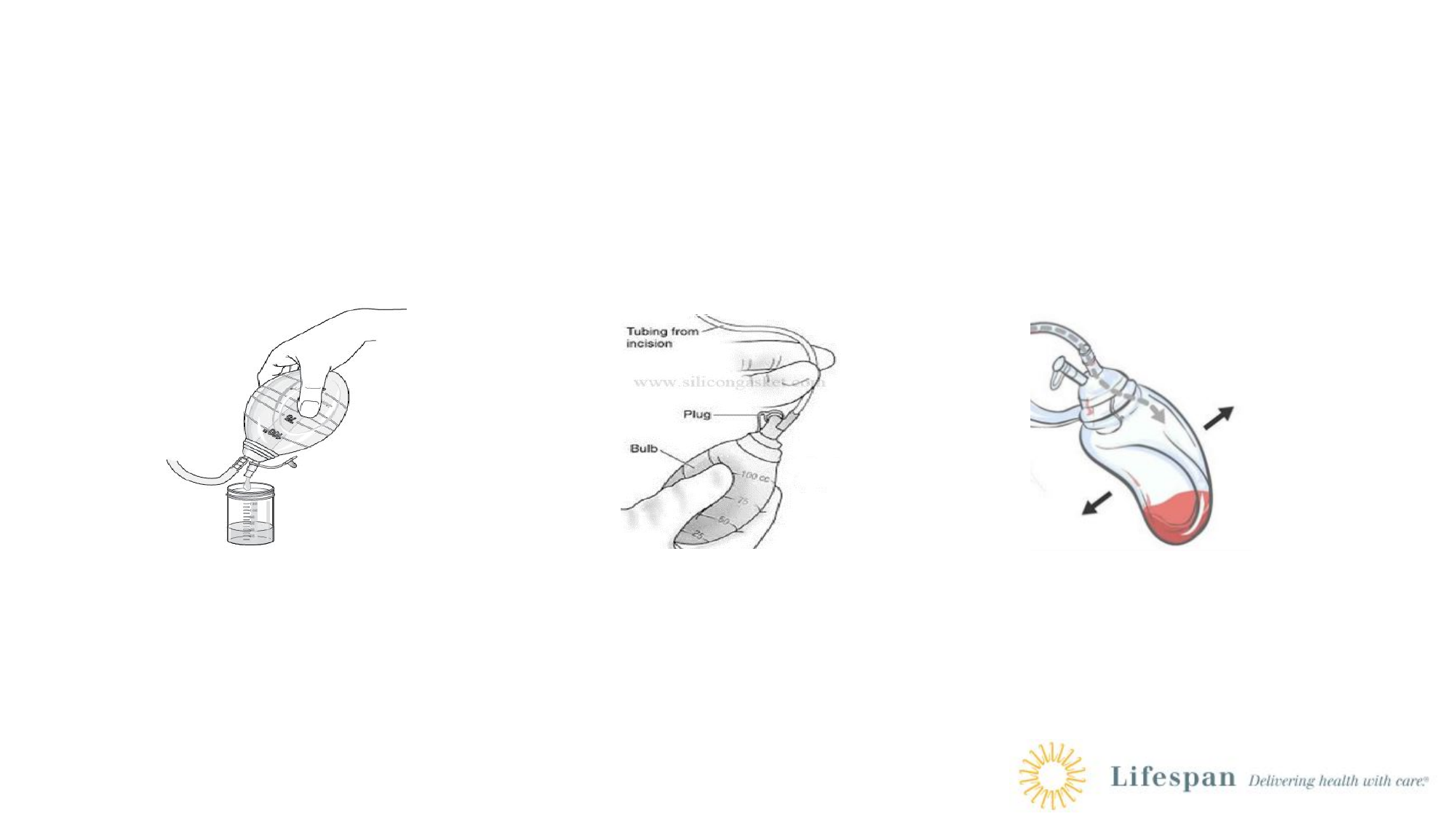

#

## Slide 13
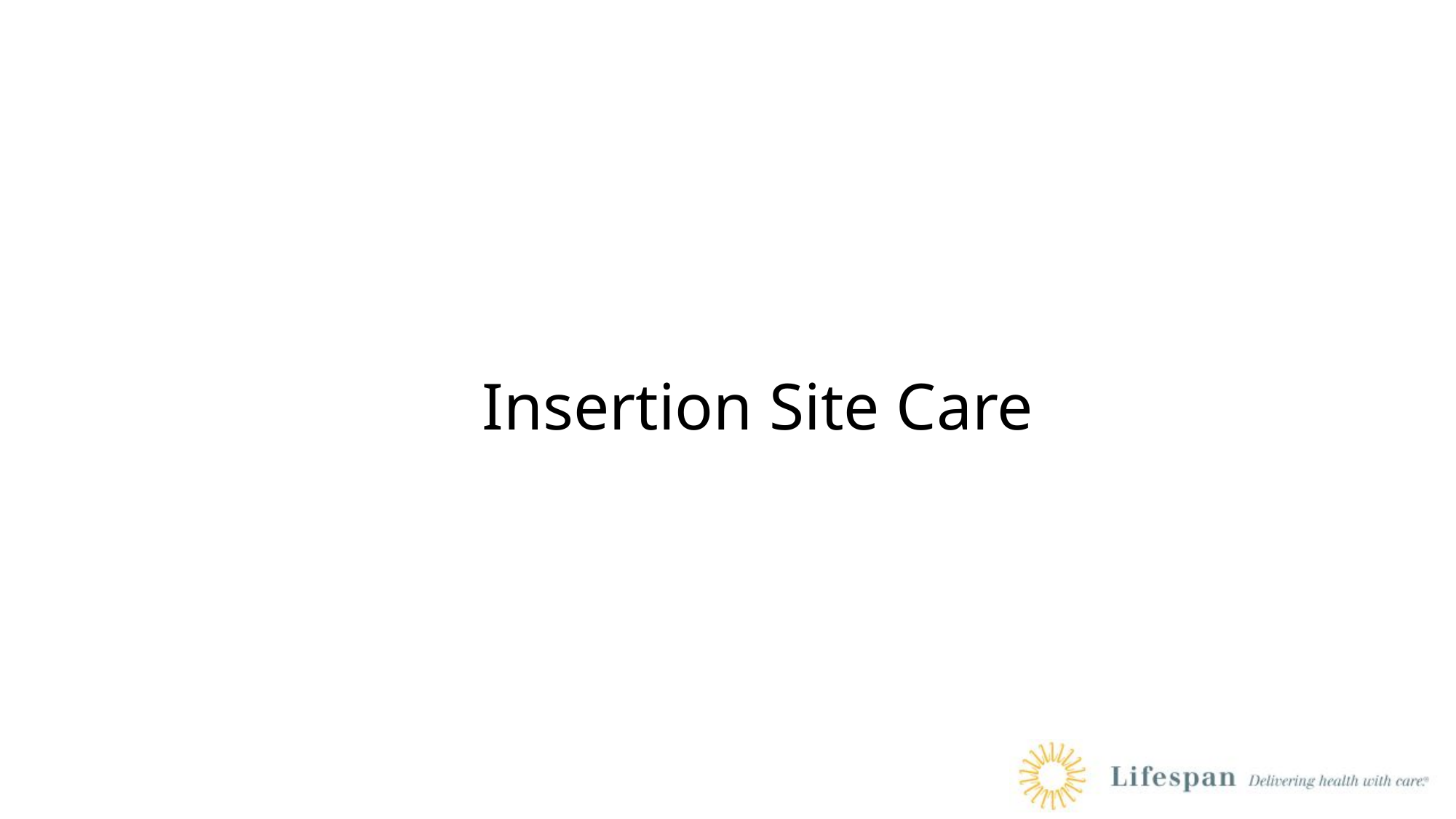

# Insertion Site Care

## Slide 14
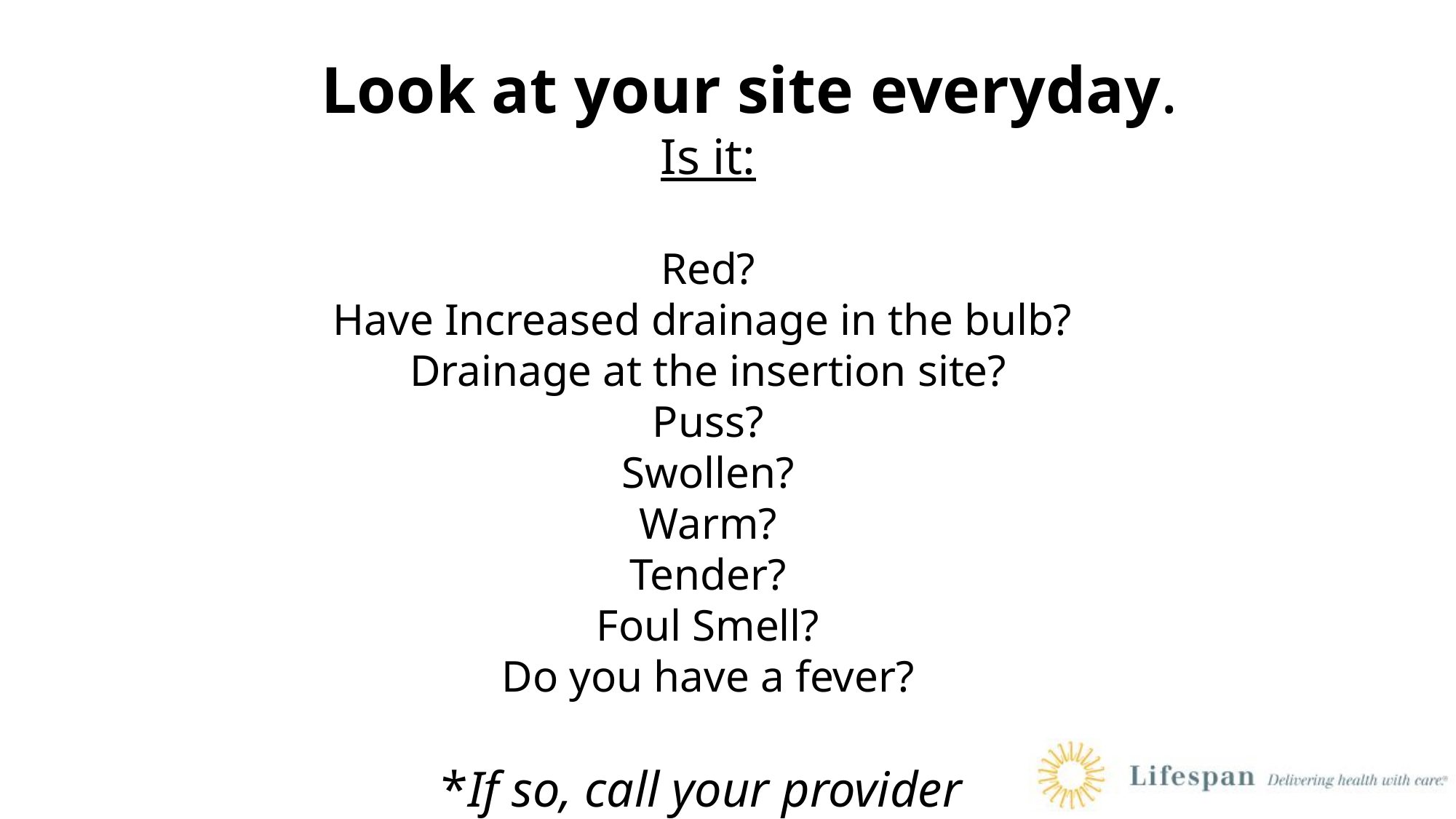

# Look at your site everyday. Is it: Red? Have Increased drainage in the bulb? Drainage at the insertion site? Puss? Swollen? Warm? Tender? Foul Smell? Do you have a fever? *If so, call your provider

## Slide 15
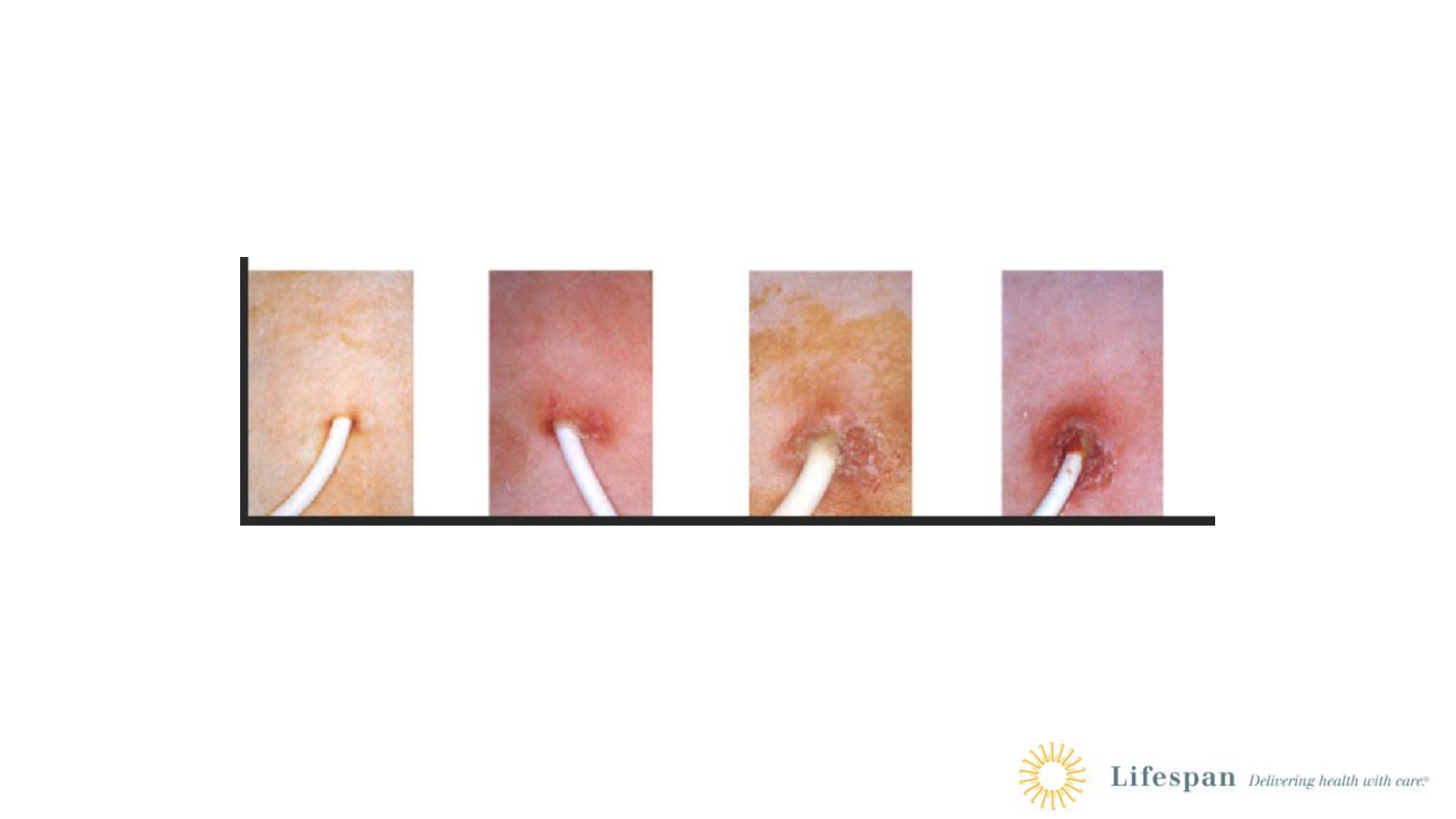

#

## Slide 16
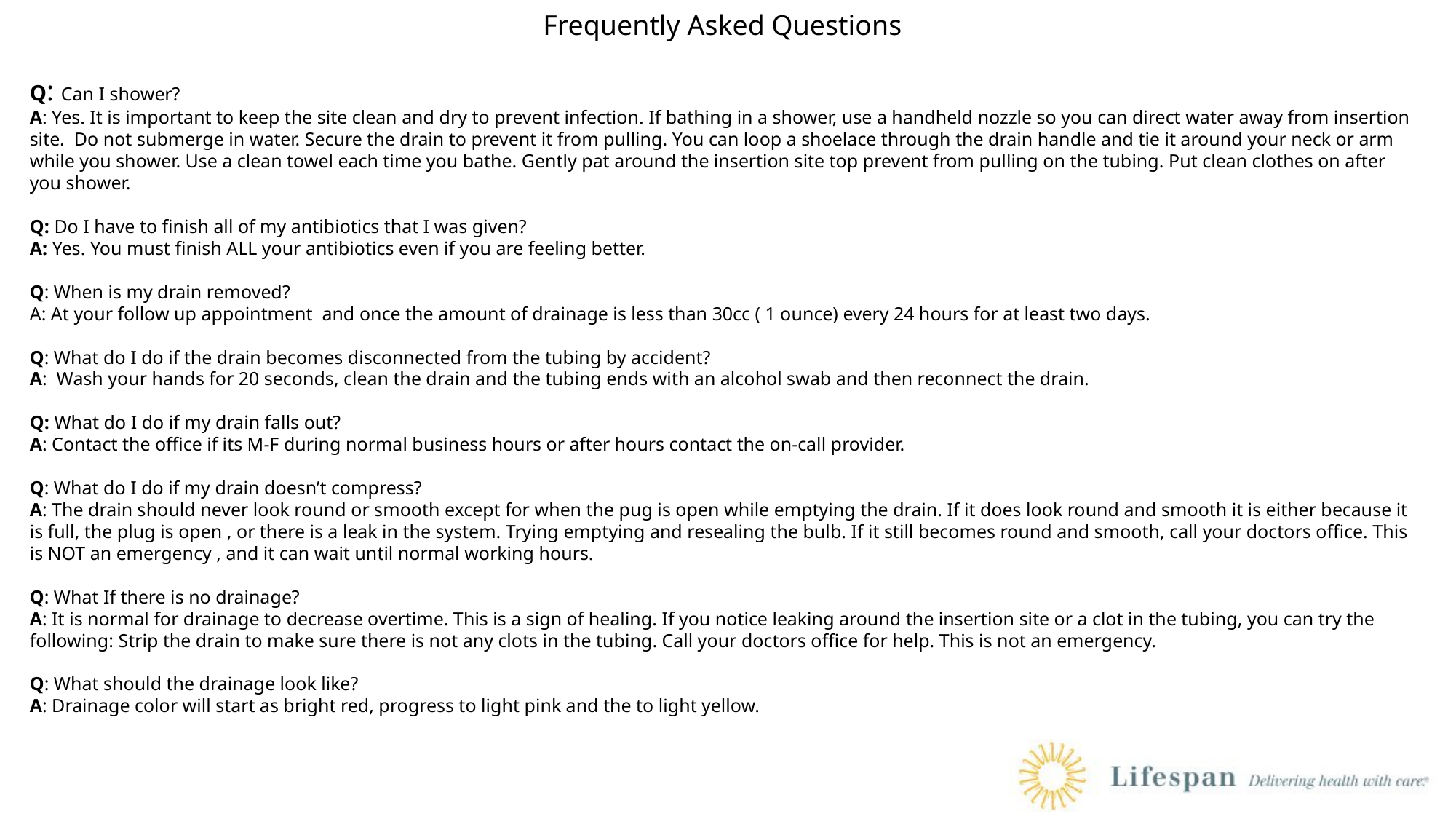

Frequently Asked Questions
Q: Can I shower?
A: Yes. It is important to keep the site clean and dry to prevent infection. If bathing in a shower, use a handheld nozzle so you can direct water away from insertion site. Do not submerge in water. Secure the drain to prevent it from pulling. You can loop a shoelace through the drain handle and tie it around your neck or arm while you shower. Use a clean towel each time you bathe. Gently pat around the insertion site top prevent from pulling on the tubing. Put clean clothes on after you shower.
Q: Do I have to finish all of my antibiotics that I was given?
A: Yes. You must finish ALL your antibiotics even if you are feeling better.
Q: When is my drain removed?
A: At your follow up appointment and once the amount of drainage is less than 30cc ( 1 ounce) every 24 hours for at least two days.
Q: What do I do if the drain becomes disconnected from the tubing by accident?
A: Wash your hands for 20 seconds, clean the drain and the tubing ends with an alcohol swab and then reconnect the drain.
Q: What do I do if my drain falls out?
A: Contact the office if its M-F during normal business hours or after hours contact the on-call provider.
Q: What do I do if my drain doesn’t compress?
A: The drain should never look round or smooth except for when the pug is open while emptying the drain. If it does look round and smooth it is either because it is full, the plug is open , or there is a leak in the system. Trying emptying and resealing the bulb. If it still becomes round and smooth, call your doctors office. This is NOT an emergency , and it can wait until normal working hours.
Q: What If there is no drainage?
A: It is normal for drainage to decrease overtime. This is a sign of healing. If you notice leaking around the insertion site or a clot in the tubing, you can try the following: Strip the drain to make sure there is not any clots in the tubing. Call your doctors office for help. This is not an emergency.
Q: What should the drainage look like?
A: Drainage color will start as bright red, progress to light pink and the to light yellow.
#

## Slide 17
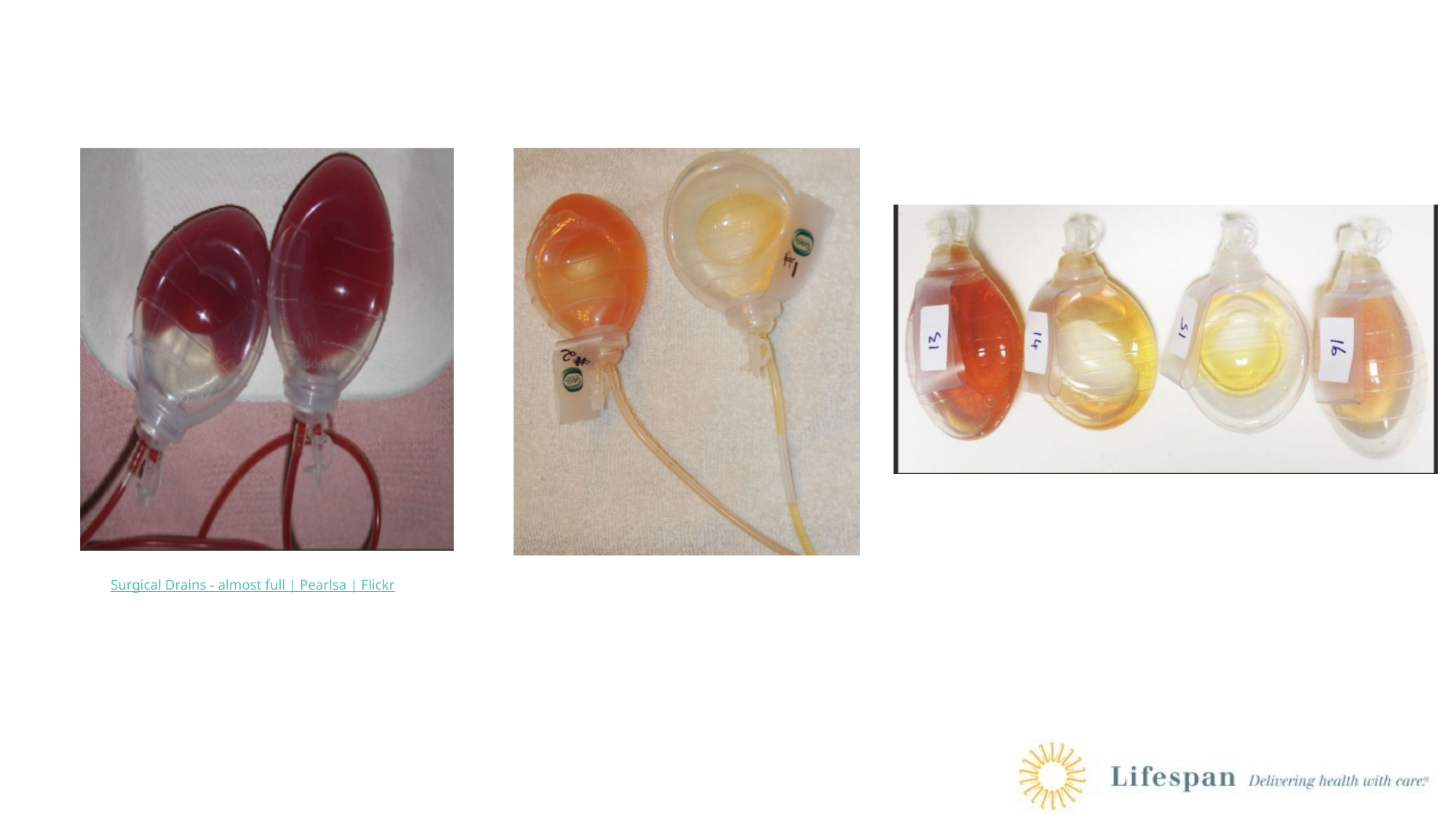

#
Surgical Drains - almost full | Pearlsa | Flickr

## Slide 18
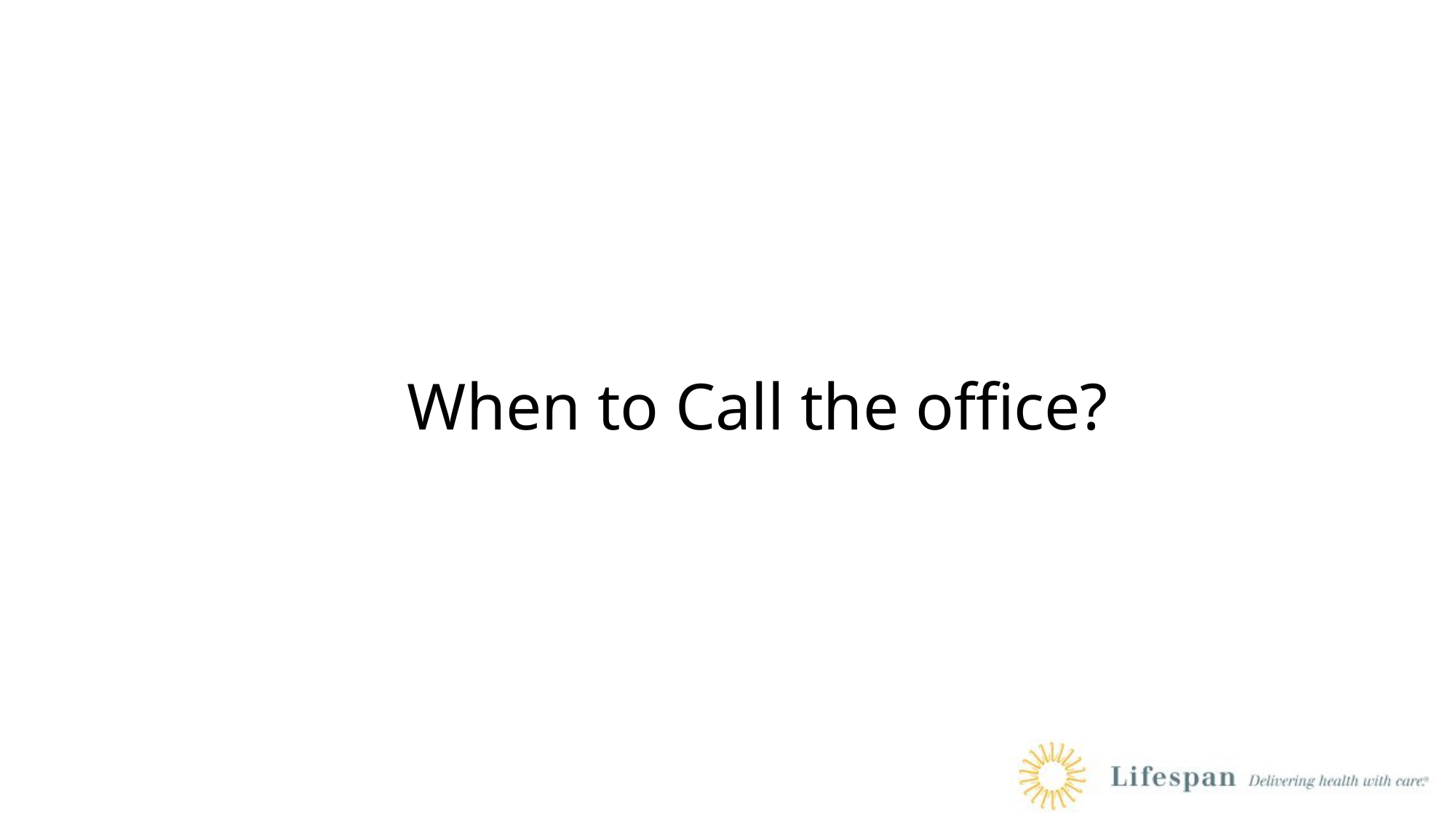

# When to Call the office?

## Slide 19
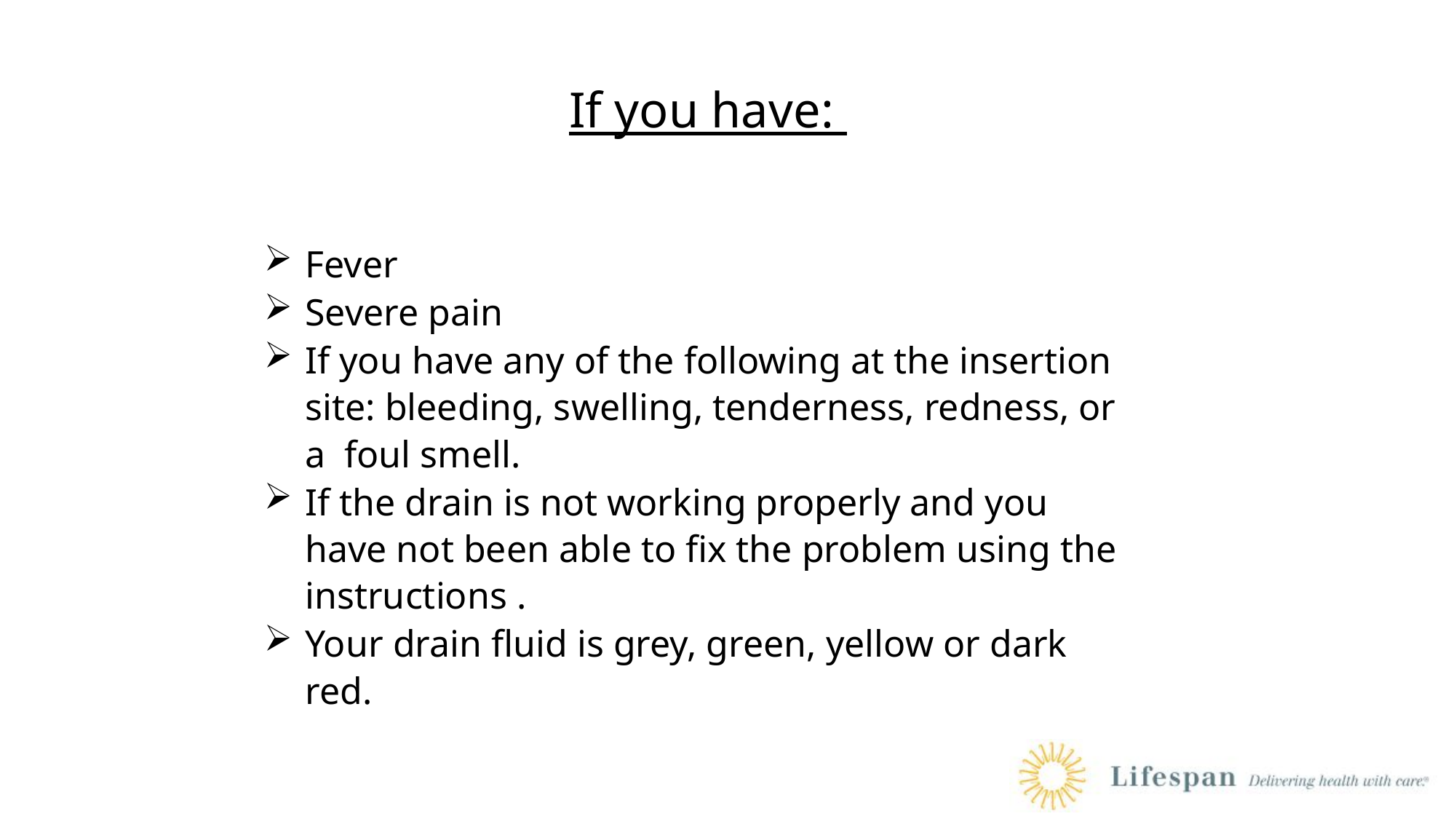

#
If you have:
Fever
Severe pain
If you have any of the following at the insertion site: bleeding, swelling, tenderness, redness, or a foul smell.
If the drain is not working properly and you have not been able to fix the problem using the instructions .
Your drain fluid is grey, green, yellow or dark red. dark red.

## Slide 20
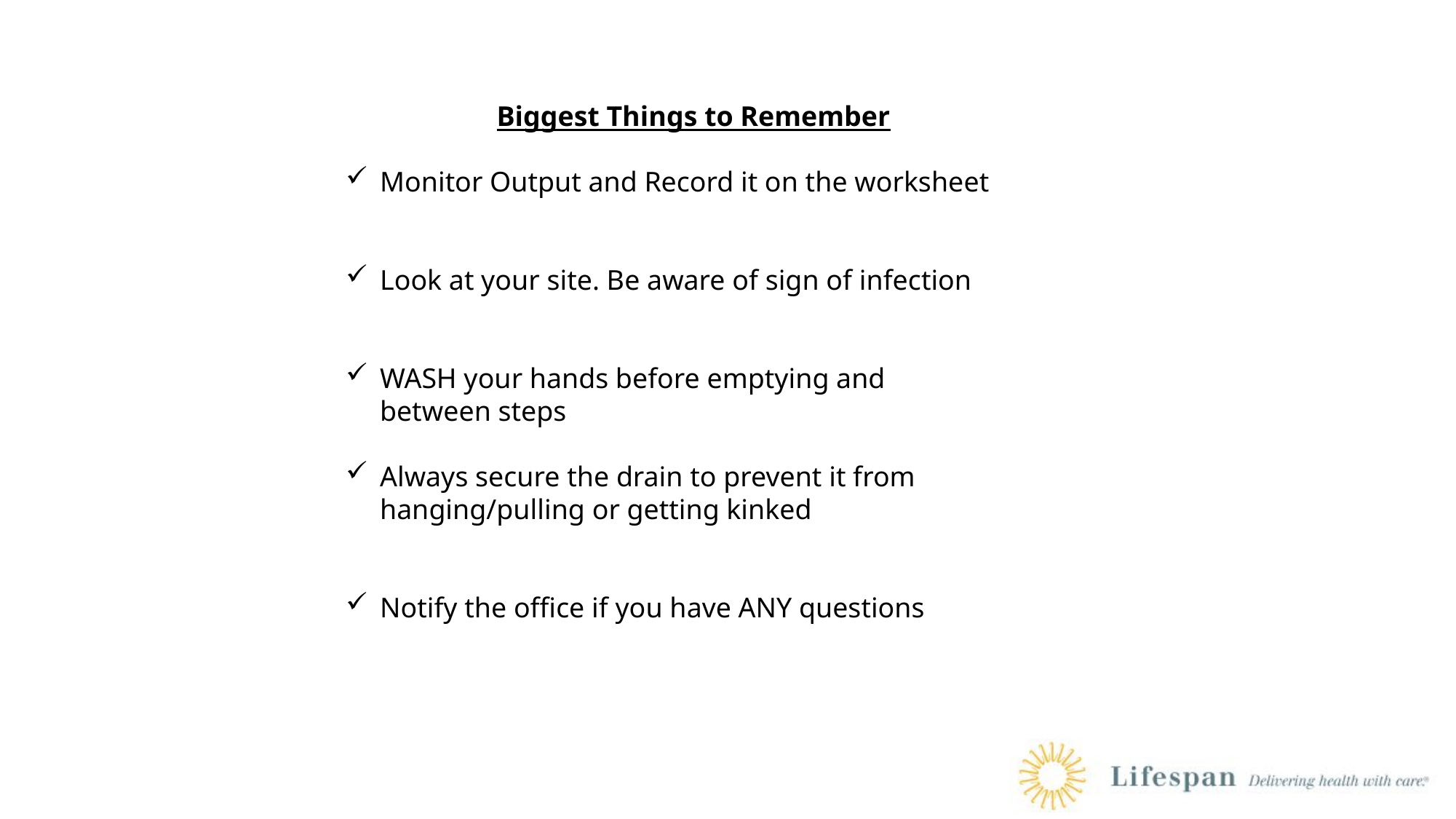

#
 Biggest Things to Remember
Monitor Output and Record it on the worksheet
Look at your site. Be aware of sign of infection
WASH your hands before emptying and between steps
Always secure the drain to prevent it from hanging/pulling or getting kinked
Notify the office if you have ANY questions

## Slide 21
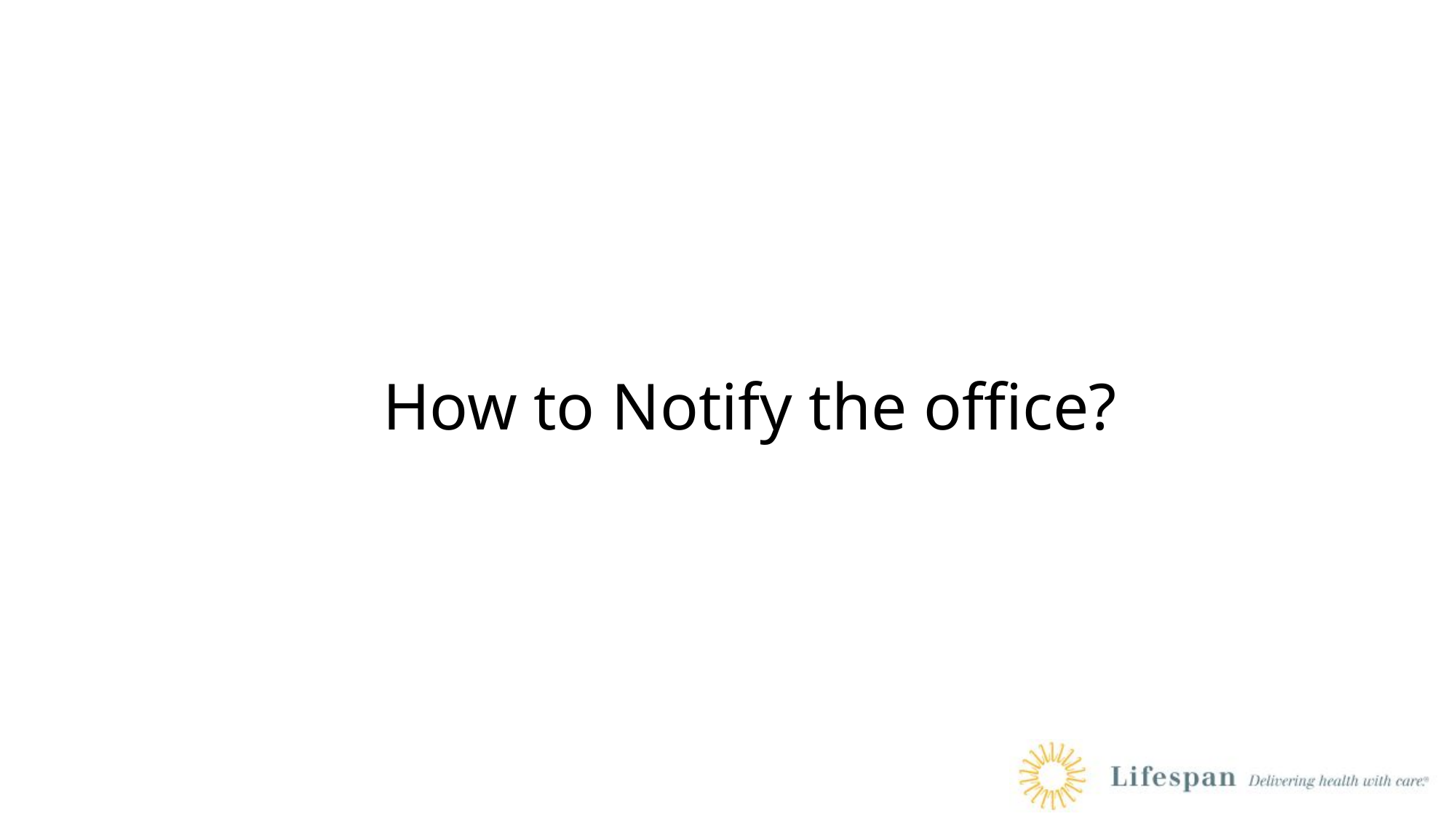

# How to Notify the office?

## Slide 22
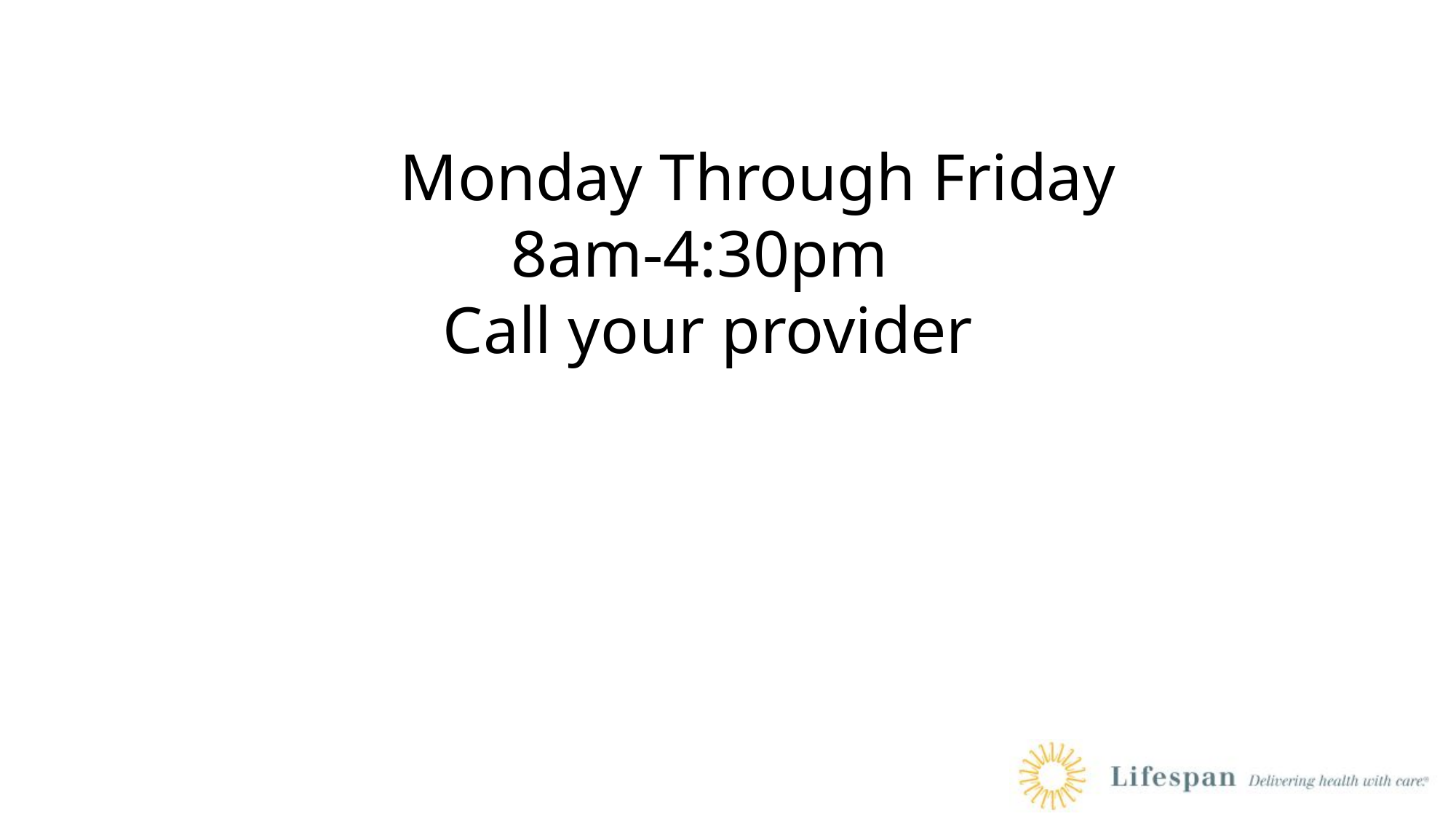

# Monday Through Friday 8am-4:30pm Call your provider
