# Supplementary material for: Surgical site infections and the discharge care of surgical drains following spinal fusions: a qualitative inquiry
Source: Antimicrob Steward Healthc Epidemiol. 2025 Oct 1;5(1):e241. doi: 10.1017/ash.2025.10152 (PMC12509140; doi:10.1017/ash.2025.10152)
Supplement: Leson et al. supplementary material 2 — Leson et al. supplementary material [file S2732494X25101526sup002.docx]

**Drain Discharge Instructions**

**General Information**

- You are being discharged with a bulb drain.
-
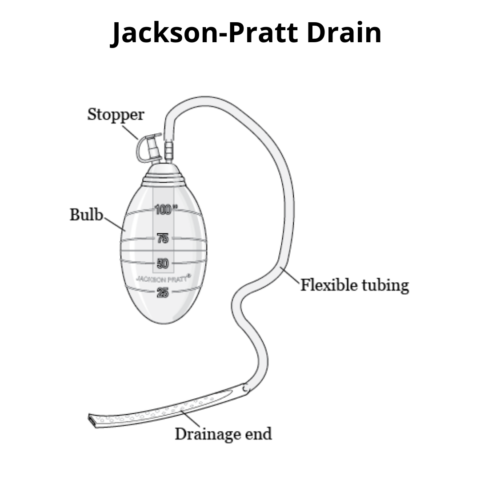
This drain will collect fluid from your surgical site.
- It is important that you keep the insertion site (the area where the drain is inserted into your body) clean and dry to prevent infection.
- There is a stitch attaching the drain to your body. The stitch will be removed when the drain is removed.
- Your nurse and these instructions will go over how to empty the drain.
- Remember, the most important way to prevent infection is to **WASH YOUR HANDS.** Whenever you are handling (touching) the drain, you should always have clean hands.
-
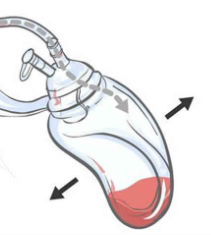
For your drain to work, it should be compressed (see picture below).

**Daily Drain Care**

- Before touching the drain or the insertion site **WASH YOUR HANDS** with soap and water for 20 seconds.
- Every day, look at the insertion site. If the area is red, swollen, warm, tender, has a foul smell, or you have a fever, you should call the doctors office.
-
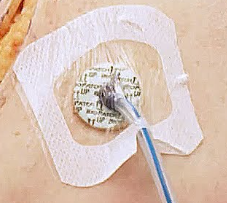

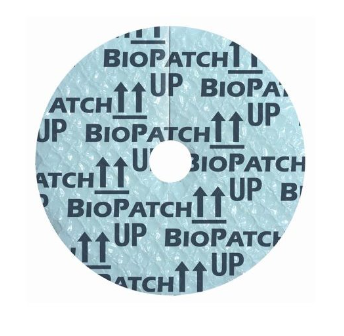
The drain should be dressed with a BioPatch at the insertion site and covered with a clear dressing. The dressing should be changed every 7 days and in-between as needed (if it becomes wet/dirty).

*This is a BioPatch. The BioPatch should be placed against the skin, around the insertion site with the word side up. It should then be covered with a clear dressing as shown in the photo above.*

- Emptying the Drain
  - Empty the drain at least two (2) times a day.
    - You might need to empty the drain more often if it is full.
    - Record the amount of fluid on your drain output worksheet
  - **Wash your hands.**
  - Open the stopper (plug) and pour the contents into a measuring cup.
  - When all the contents have been emptied, squeeze the drain bulb and put the stopper (plug) back on the drain.
  - Wash your hands.
  -
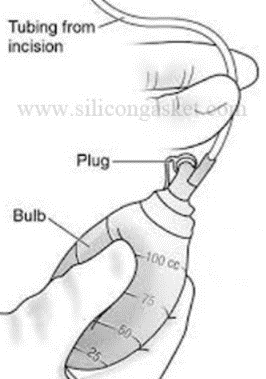

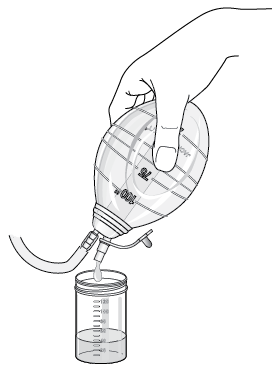
Measure the amount of fluid that you emptied from the drain and record it on the drain output worksheet.
  - The drain will be removed once the amount of drainage is less than 30cc (about 1 ounce) every 24 hours for at least 2 consecutive days.
- “Stripping” the Drain
  - You should strip the drain a few times a day. “Stripping the drain” prevents the drain from getting clogged.
  - Wash your hands.
  - Use one hand to tightly secure the drain where it enters your skin. Use the other hand's forefinger and thumb to squeeze the tubing and pull towards the bulb.
  - The fluid from the tubing will move down the tubing and into the drain bulb.
- Showering/Bathing
  - It is important to keep the insertion site clean and dry to prevent infection.
  - **If bathing in a shower, make sure you have a handheld nozzle so you are able to direct water away from the insertion site.**
  - Do not submerge your incision in the water.
  - When you shower, secure your drains to prevent them from pulling.
    - You can loop a shoelace through the drain handle and tie it around your neck or arm to wear as a necklace while you shower.
  - Dry off with a CLEAN TOWEL each time you shower/bathe.
    - Gently pat around the insertion site to prevent pulling on the line.
  - Put on clean clothes after you shower.

*If you have an open wound around your incision, sponge bathe only unless you have discussed other bathing options with your provider.*

- Medications
  - If you have been prescribed antibiotics, take ALL of your medication. Do not stop taking your medication even if you think you are feeling better. You must finish your entire course of antibiotics.

**Potential Problems and How to Solve Them**

- What to do if… the drain becomes disconnected from the tubing
  - NEVER disconnect the drain bulb from the drain tubing!
  - If your drain becomes disconnected from the tubing by accident, wash your hands with soap and water for 20 seconds, clean the drain and the tubing ends with an alcohol swab and then reconnect the drain.
- What to do if…the drain does not stay compressed.
  - The drain should never look round and smooth (like a “hand grenade”) except when the plug is open while emptying the drain. If it does look round and smooth, it is either full, the plug is open, or there is a leak in the system.
  - Try emptying and resealing the bulb. If it still becomes round and smooth, call your doctor’s office (this is not an emergency, and it can wait until working hours).
- What to do if…there is no drainage in your drain
  - It is normal for drainage to decrease over time. This is a sign of healing. If you notice leaking around the insertion site or a clot in the tubing you can try the following:
    - Strip the drain to make sure there are not any clots in the tubing.
    - Call your doctors office for help (this is not an emergency)

**When to call:**

1. Call if you have bleeding, severe pain, swelling, redness around the drain site, or fever.
2. Call if the drain is not working properly and you have not been able to fix the problem using the instructions above.
3. Your drain fluid should look clear yellow to dark pink. Call your doctor if it is grey, green, yellow, or dark red.
4. Call if you have any questions.
5. Your doctor/provider is: ______________. Office phone number: _____________.

**Drain output worksheet:**

|  |  |  |  |  |  |  |  |  |  |  |  |  |  |  |  |
| --- | --- | --- | --- | --- | --- | --- | --- | --- | --- | --- | --- | --- | --- | --- | --- |
| **Date:** | |  |  |  |  |  |  |  |  |  |  |  |  |  |  |
| **Drain 1** | **AM** |  |  |  |  |  |  |  |  |  |  |  |  |  |  |
|  | **PM** |  |  |  |  |  |  |  |  |  |  |  |  |  |  |
|  | **Total** |  |  |  |  |  |  |  |  |  |  |  |  |  |  |
|  |  |  |  |  |  |  |  |  |  |  |  |  |  |  |  |
|  | **AM** |  |  |  |  |  |  |  |  |  |  |  |  |  |  |
|  | **PM** |  |  |  |  |  |  |  |  |  |  |  |  |  |  |
|  | **Total** |  |  |  |  |  |  |  |  |  |  |  |  |  |  |
